# Neutrophil/lymphocyte ratio predicts in‐hospital complications in Takotsubo syndrome. Results from a prospective multi‐center registry

**DOI:** 10.1002/clc.23442

**Published:** 2020-08-08

**Authors:** Francesco Santoro, Francesca Guastafierro, Tecla Zimotti, Adriana Mallardi, Alessandra Leopizzi, Michele Cannone, Matteo Di Biase, Natale Daniele Brunetti

**Affiliations:** ^1^ Department of Medical and Surgical Sciences, Foggia, Bonomo Hospital Andria Italy; ^2^ Department of Medical and Surgical Sciences University of Foggia Foggia Italy; ^3^ Cardiology Department Bonomo Hospital Andria Italy

**Keywords:** apical ballooning syndrome, complications, hemogram, neutrophil/lymphocyte ratio, Takotsubo syndrome

## Abstract

**Background:**

Several hematological indices including subtypes of leukocytes populations have been associated with cardiovascular outcome. Takotsubo syndrome (TTS) is a form of acute heart failure syndrome featured by several in‐hospital complications (IHCs).

**Hypothesis:**

Hematological indices at admission may predict IHCs in TTS patients.

**Methods:**

One hundred and sixty consecutive patients with TTS were enrolled in a multicenter prospective registry. Clinical data, admission hemogram, and IHCs were recorded.

**Results:**

Incidence of IHCs was 37%, including pulmonary edema 9%, cardiogenic shock 9%, need of invasive ventilation 10%, death 8%, stroke 2.5%, and left ventricular thrombi 6%.

Patients with IHCs were older, more frequently male, with physical stressor‐induced TTS, lower left ventricular ejection fraction at admission. Neutrophil/lymphocyte ratio (NLr) (12 ± 12 vs 7 ± 8, *P* = .002) and white blood cells/mean platelet volume ratio (1.2 ± 0.5 vs 1.0 ± 0.5, *P* = .03) at admission were significantly higher in patients with IHCs.

NLr values were predictor of IHCs (Odds ratios [OR] 1.07, 95% CI 1.03‐1.11, *P* < .01). When stratified according to NLr into tertiles, the rate of IHCs was from first to third tertile was, respectively, 22%, 31%, and 58%. NLr values in the higher tertile were independent predictors of IHCs even at multivariate analysis (OR 3.7, 95% CI 1.5‐9.4, *P* < .01).

NLr values higher than 5 were able to predict IHCs with a sensitivity of 82% and specificity of 58%; negative predictive power was 84% (area under the ROC curve 0.73).

**Conclusions:**

NLr is an independent predictor of IHCs in patients admitted with TTS. Admission hemogram may represent a potential tool for prediction of IHCs in TTS.

## 1 INTRODUCTION

Takotsubo syndrome (TTS) is a form of acute heart failure featured by transient left ventricular dysfunction that can mimic acute myocardial infarction.^1^ It mostly affects postmenopausal women after either a physical or an emotional stress. Recent studies showed that TTS is featured by high rate of in‐hospital complications (IHCs), mainly cardiovascular^2^ and adverse events at long term.^3^

Several algorithms based on the use of clinical and echocardiographic parameters have been proposed for in‐hospital risk stratification in TTS patients^2^; less is known, however, on the prognostic role of hematological indices in TTS.

In previous studies, the neutrophil/lymphocyte ratio (NLr) has been related with the occurrence of in‐hospital cardiovascular complications and mayor cardiovascular adverse events in patients with acute coronary syndrome (ACS)^4^.^5^ Aim of this study was therefore to evaluate whether the assessment of a hematological index, such as NLr at admission could be useful to predict clinical outcomes during hospitalization in TTS patients.

## 2 METHODS

### 2.1 Study population

The study included 160 consecutive patients with TTS enrolled from April 2009 to May 2018 in two Italian hospitals (Riuniti University Hospital of Foggia, Apulia, Italy and Bonomo Hospital, Andria, Apulia, Italy). The entire study population fulfilled the 2008 revised Mayo Clinic diagnostic criteria: (a) transient hypokinesis, dyskinesis, or akinesis of the left ventricular (LV) apical and/or midventricular or basal segments extending beyond a single epicardial vessel distribution territory; (b) absence of significant obstructive coronary artery disease explaining the extent of wall motion abnormalities or angiographic evidence of acute plaque rupture; (c) new electrocardiogram (ECG) abnormalities (either ST‐segment elevation and/or T‐wave inversion) or modest elevation in the cardiac troponin levels; (d) absence of pheochromocytoma or myocarditis or intracranial hemorrhage.^6^

The study was held according to declaration of Helsinki and approved by local ethics committees; all participants provided a written informed consent.

### 2.2 Clinical examination and echocardiography

All patients underwent a clinical examination and a detailed anamnesis was collected including: age, gender, and kind of triggering stressor. Medical history, including history of neurologic disorders (cerebrovascular accidents, neurodegenerative disorders, and epilepsy), was also recorded.

A two‐dimensional Doppler Echocardiographic examination at admission, at third day, and at discharge was performed. The left ventricular ejection fraction (LVEF) was calculated using the Simpson method from the apical four‐chamber and two‐chamber view. Coronary angiography was performed in all patients at admission.

### 2.3 Blood tests

The complete blood cell count was performed after blood samples with K2EDTA tubes (Terumo Europe NV, Leuven, Belgium), which were always collected at the time of admission. The analysis was performed with the fully automated hematological analyzer Advia 2120 (Siemens Healthcare Diagnostics, Tarrytown NY). The local reference ranges are 150 to 400 × 10^9^/L for the platelet count, 4.3 to 10.0 × 10^9^/L for total white blood cells, 2.0 to 7.0 × 10^9^/L for neutrophils, and 0.95 to 4.5 × 10^9^/L for lymphocytes. The entire investigation was carried out using the same analyzer and the quality of results was validated throughout the study period by regular internal quality control procedures and participation in an external quality assessment scheme.

The values of NLr, white blood cell count/mean platelet volume (MPV) ratio, MPV/platelets ratio, and platelet count/lymphocyte ratio were, respectively, calculated by dividing the absolute number of each variable obtained in the same blood sample collected at admission.

### 2.4 Outcomes

The primary clinical endpoint was occurrence of IHCs including overall mortality, pulmonary oedema, need of invasive ventilation, cardiogenic shock, stroke, and LV thrombosis.

### 2.5 Statistical analysis

Continuous variables were expressed as mean ± SD and compared with Student *t* test or Mann‐Whitney *U* test as required. Categorical variables were presented as percentages and compared with *χ* ^2^ or Fisher test as required. The Kolmogorov‐Smirnov test was used to identify variables with normal distribution.

Logistic regression analysis was used to identify predictors of outcome. Forward multivariable logistic regression analysis was used for correcting for principal confounders. Predictors at univariable logistic regression with a significance value ≤0.01 were included in multivariable analysis. Odds ratios (OR) with 95% confidence interval (CI) were calculated. Linear correlations were determined by measuring the Pearson's correlation coefficient.

A *P* < .05 was considered as statistically significant.

## 3 RESULTS

### 3.1 Patient characteristics and in‐hospital complications

Mean age was 74 ± 12 years, mean LVEF 36 ± 8%, 11% (17 patients) were male (Table 1). Rate of IHCs occurrence was 37%, including pulmonary edema 9%, cardiogenic shock 9%, need of invasive ventilation 10%, death 8%, stroke 2.5%, and LV thrombi 6%.

**TABLE 1:** Baseline features according to occurrence of IHCs

Variable	IHCs	No IHCs	*P* value
Age	78 ± 9	73 ± 13	.01
Male	18%	6%	.01
Hypertension	72%	80%	.22
Dyslipidemia	52%	45%	.39
Obesity	28%	32%	.64
Smoker	15%	13%	.71
Diabetes	34%	21%	.07
History of previous neurological disorders	40%	17%	.01
History of rheumatic disorders	5%	8%	.53
COPD	35%	27%	.27
Clinical presentation			
No chest pain	42%	25%	.02
Typical chest pain	32%	53%	.01
Atypical chest pain	10%	22%	.06
Dyspnoea	41%	41%	.96
Triggering stressor			
Emotional stressor	17%	30%	.06
Physical stressor	61%	42%	.02
Infectious/inflammatory	26%	12%	.03
Non‐infectious/inflammatory	35%	30%	.49
No stressor	22%	28%	.40
Echocardiographic features			
LVEF at admission	33%	37%	.01
Apical ballooning	98%	92%	.09
Midventricular ballooning	2%	8%	.09
Basal ballooning	0	0	—
ECG features at admission			
Negative T waves	48%	50%	.83
ST elevation	52%	43%	.28
Long QT interval	42%	41%	.85

Abbreviations: IHC, in‐hospital cardiovascular complications; COPD, chronic obstructive pulmonary disease; LVEF, left ventricular ejection fraction; ECG, electrocardiogram.

Patients with IHCs were older (78 ± 9 vs 72 ± 13 years, *P* < .01), more frequently male (24% vs 6%, *P* = .01), more frequently presented a physical stressor (64% vs 43%, *P* = .02), with higher prevalence of infective/inflammatory triggers (26 vs 12% *P* = .03) or absence of chest pain (42% vs 25%, *P* = .01), and a lower LVEF at admission (33 ± 7% vs 37 ± 8%, *P* = .01) (Table 1).

### 3.2 Hemogram at admission

Patients with IHCs presented higher values of white blood cells (13 ± 5 vs 11 ± 5 10^3^/μL, *P* = 0.01), neutrophils (13 ± 15 vs 9 ± 9 10^3^/μL, *P* = .02), and lower values of eosinophils (0.03 ± 0.1vs 0.08 ± 0.1 10^3^/μL *P* = <.01). NLr (12 ± 12 vs 7 ± 8; *P* = .002) and white blood cells/MPV ratio (1.2 ± 0.5 vs 1.0 ± 0.5, *P* = .03) were significantly higher in patients with IHCs (Table 2).

**TABLE 2:** Admission hemogram parameters according to occurrence of IHCs

Variable	IHCs	No IHCs	*P* value
RBC × 10^6^/μL	4.5 ± 1.5	4.4 ± 0.7	.47
HB, g/dL	12 ± 2	12 ± 2	.98
HCT, %	38 ± 6	37 ± 5	.50
MCV, fL	88 ± 6	87 ± 8	.18
MCH, pg	29 ± 3	29 ± 3	.58
MCHC, g/dL	33 ± 2	33 ± 1	.09
RDW, %	15 ± 1	15 ± 2	.33
PLT 10^3^/μL	247 ± 87	254 ± 124	.72
MPV, fL	11 ± 1	11 ± 1	.35
PCT, %	0.27 ± 0.09	0.27 ± 0.12	.86
PDW, fL	15 ± 3	14 ± 3	.56
NEUTR × 10^3^/μL	13 ± 15	9 ± 9	.02
LYN × 10^3^/μL	1.7 ± 1.6	1.8 ± 1.2	.63
EOS × 10^3^/μL	0.03 ± 0.1	0.08 ± 0.1	<.01
BAS × 10^3^/μL	0.07 ± 0.34	0.02 ± 0.02	.19
WBC × 10^3^/μL	13 ± 5	11 ± 5	.01
WBC/MPV ratio	1.16 ± 0.48	0.97 ± 0.48	.03
NEUT/LYN ratio	12 ± 12	7 ± 8	.01
MPV/PLT ratio	0.05 ± 0.02	0.05 ± 0.03	.93
PLT/LYN ratio	267 ± 222	208 ± 266	.14

Abbreviations: BAS, basophil; EOS, eosinophil; HB, hemoglobin; HCT, hematocrit; IHC, in‐hospital cardiovascular complications; Lyn, lymphocyte; MCH, mean corpuscular hemoglobin; MCHC, mean corpuscular hemoglobin concentration; MCV, mean corpuscular volume; Neutr, neutrophil; PDW, platelet distribution width; PLT, platelet; RBC, red blood cell; RDW, red cells distribution width; WBC, white blood cell.

### 3.3 Predictive value of NLr

**FIGURE 1:**
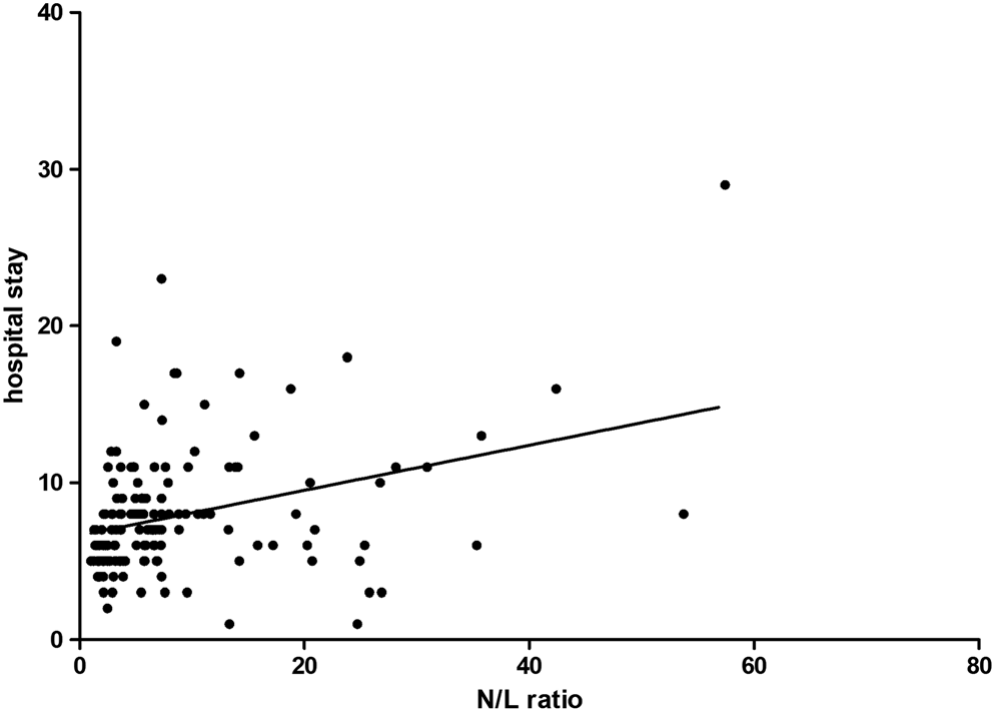
Linear correlation between NLr and hospital stay duration (days). N/Lr, neutrophil/lymphocyte ratio

**FIGURE 2:**
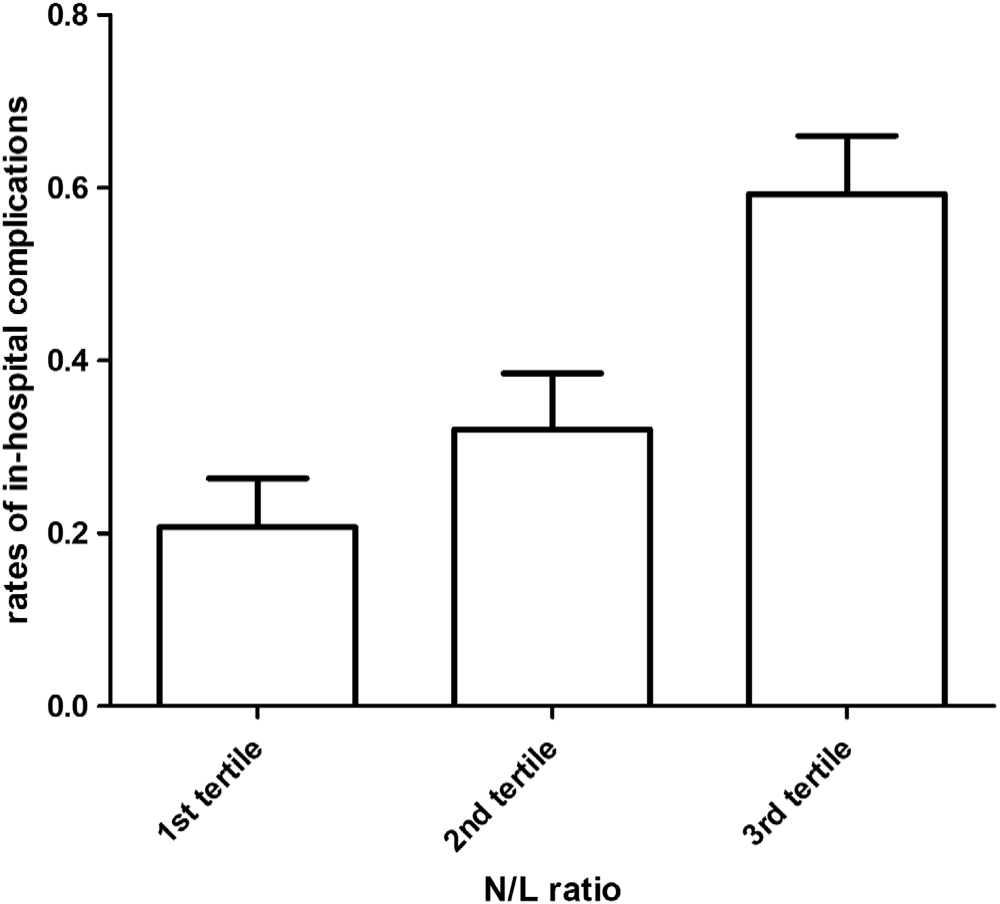
In‐hospital cardiovascular events stratified according to neutrophils/lymphocytes ratio in tertiles

NLr values were significant predictors of IHCs (OR 1.07, 95% CI 1.03‐1.11, *P* < .01) and significantly correlated with hospital stay duration (r = 0.34, *P* < .001) (Figure 1). When stratified according NLr tertiles, the rate of IHCs occurrence was 22%, 31%, and 58% (Figure 2). NLr values in the higher tertile were independent predictors of IHCs at multivariate forward stepwise logistic regression analysis (OR 3.7, 95% CI 1.5‐9.4, *P* < .01) (Table 3).

**TABLE 3:** Univariate and multivariate forward stepwise logistic regression analysis: predictors of in‐hospital cardiovascular complications

	Univariate OR	95% CI	*P*	Multivariate OR	95% CI	*P*
NLr (upper tertile vs lower)	5.4	2.3‐12.7	.0001	3.7	1.5‐9.4	.0051
Systolic pressure at admission (mm Hg)	0.96	0.94‐0.98	<.0001	1.0	0.9‐1.0	.0029
Neurologic disease	3.3	1.6‐7.0	.0015			
Age (y)	1.05	1.01‐1.09	.0050			
Male gender	3.5	1.2‐10.0	.0203			
Eosinophils (10^3^/μL)	0.0003	<0.0001‐0.1	.0063			
LVEF at admission	0.0022	<0.0001‐0.2	.0059			
WBC (10^3^/μL)	1.1	1.02‐1.17	.0146			
Typical chest pain	0.4	0.2‐0.8	.0118			

Abbreviations: LVEF, left ventricular ejection fraction; NLr, neutrophil/lymphocyte ratio; OR, odds ratio; WBC, white blood cells.

**FIGURE 3:**
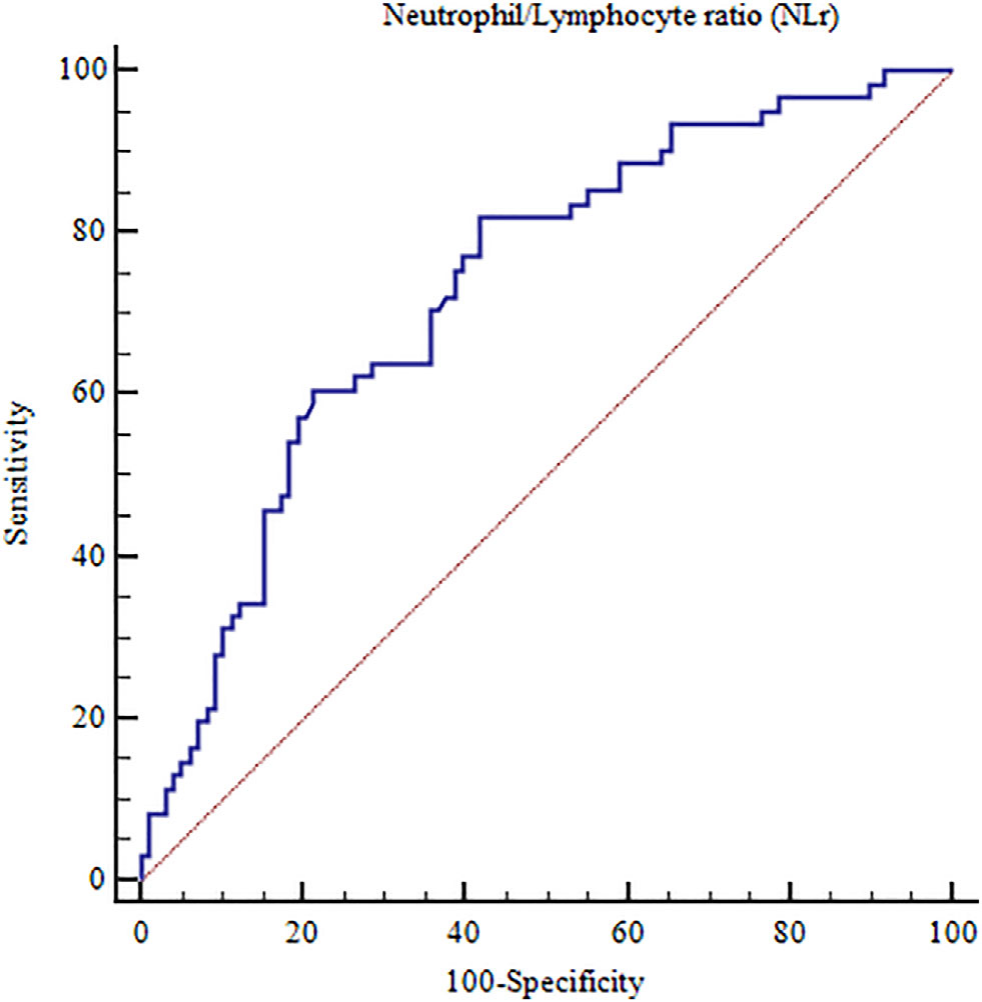
Area under the ROC curve of neutrophils/lymphocytes ratio and occurrence of in‐hospital complications in patients with Takotsubo Syndrome

NLr values >5 were able to predict the occurrence of IHCs with a sensitivity of 82% and a specificity of 58%; negative predictive power was 84%, positive predictive power 54%, accuracy 63%. NLr area under the ROC curve was 0.73 (95% CI 0.65‐0.79, *P* < .01) (Figure 3).

## 4 DISCUSSION

To the best of our knowledge, this is one of the first studies showing possible correlations between hemogram parameters and IHCs in TTS. Main findings of the study are the followings:

1) Patients that experienced IHCs presented higher values of white blood cells and neutrophils at admission;

2) NLr is an independent predictor of IHCs;

3) When stratified according to NLr tertiles, values in the higher tertile were independent predictors of IHCs.

TTS has been for a long time considered a benign disease, because of the completely reversible nature of the distinct wall motion abnormalities. However, several clinical studies have shown that TTS is a form of acute heart failure that can be associated with life‐threatening complications.^7^ Therefore, an early risk stratification with simple and fast clinical tools is crucial for clinical management of these patients.

The role of hemogram parameters in cardiovascular diseases has been extensively studied and a relationship between several inflammatory markers and cardiovascular diseases has been established mainly in the context of coronary artery disease.^8^ Recent studies found that several subtypes of leukocytes compared with the total leukocyte count and specific subtype, including neutrophils, lymphocytes, and monocytes, have a better predictive value for cardiovascular events.^4^, ^5^, ^9^ Lymphocytes regulate the inflammatory response and have an anti‐atherosclerotic role in which regulatory T‐cell, a subclass of lymphocyte, may have an inhibitory effect on atherosclerosis.^10^ Lymphocyte populations and population's ratios may be associated with early clinical presentation of coronary artery disease^11^ or poor clinical outcome.^12^

Neutrophils represent the first type of white cells that is generally observed, following damage, in myocardial tissue.^13^ They also play an important role in the process of destabilization of the atherosclerotic plaque. Neutrophil count, however, is easily affected by individual variables, such as blood volume. Neutrophils secrete inflammatory mediators that can lead to vascular wall degeneration.^14^ Moreover, in an experimental study, Kim et al^15^ found that catecholamines may increase the neutrophil (PMN)‐dependent inflammatory response to cell damage. Indeed, prolonged systemic exposure of epinephrine resulted in persistent PMN trafficking.

High levels of white blood cells have been associated with an increased risk of mortality in patients with ACS.^16^ In any case, the total number of circulating leukocytes is the result of the combination of several variables, including genetics, gender, and age. A previous study also showed that a low lymphocyte count served as an early marker of physiologic stress and systemic collapse secondary to myocardial ischemia mediated by cortisol release.^17^ Increased cortisol and catecholamine levels result in a reduction in the relative level of lymphocytes.^18^, ^19^

Platelets levels in term of platelet counts and MPV may be higher in ACS as a result and a precipitating factor of inflammatory response. Megakaryocyte could be stimulated by several inflammatory mediators and present accelerated proliferation and platelet‐production. On the other hand, platelets can release mediators that promote the progression of atherosclerosis^20^.^21^ As a matter of fact, platelet/lymphocyte ratio and white blood cells/MPV ratio have been found to be predictors of in‐hospital adverse events during ACS^22^.^23^ In TTS patients, Pirzer el al found that expression of CD62P on platelets at hospital admission was lower in TTS patients vs acute myocardial infarction^24^; Nunez‐Gil et al, however, found no differences regarding platelet reactivity between the two populations.^25^ In the present study white blood cells/MPV ratio was statistically higher in TTS patients with IHCs, while no differences were found in term of platelet/lymphocyte ratio.

A systemic inflammatory activation can be observed in TTS as well as in ACS. We previously found that, in the acute and sub‐acute phase of TTS, serum levels of anti‐inflammatory interleukins (IL‐2, IL‐4, IL‐10) are higher when compared with ACS patients,^26^ suggesting a potential chronic inflammation among these patients. However, during the acute phase of TTS, increased levels of catecholamines through IL‐6 mechanism may result into persistent neutrophil inflammatory response to cell damage and relative reduction of lymphocytes levels.^27^

Murakami et al,^28^ in a Japanese registry of 107 patients with TTS, previously found that white blood cell count and brain natriuretic peptide were independent predictors of IHCs. NT‐pro‐BNP levels higher than median were found to be associated with 30‐day major adverse cardiovascular events.^29^ Additional clinical and echocardiographic parameters associated with IHCs in previous TTS cohorts were right ventricular involvement, LVEF, male sex, history of neurological disorder, and diabetes.^2^, ^30^ Recently, physical triggers have also been associated with a worse in and out‐of‐hospital outcome, especially in case of inflammatory response.^31^ These data are in line with the present study where patients with IHCs had a higher prevalence of physical triggers, mainly inflammatory.

When compared to other parameters, NLr predicted adverse events in ACS. In a meta‐analysis on 10 245 patients with ST‐elevation acute myocardial infarction after percutaneous angioplasty, Zhang et al ^32^ found a significant association between NLr and both hospitalization and adverse events including angina pectoris, heart failure, and major adverse cardiovascular events. The ratio between neutrophil and lymphocyte may thus reflect this inflammatory mechanism and represent a simple tool for clinicians for risk stratification of IHCs.

The question whether lymphocyte anomalies are induced by catecholamine surge or myocardial dysfunction, however, still remains unresolved, deserving further future investigations.

## 5 LIMITATIONS

These are preliminary data to be confirmed in larger cohorts of patients. Further and more adequately powered prospective studies are warranted to clarify the assay standardization, the optimal cutoff, and the prognostic value of NLr in association with other biomarkers and clinical scores.

Prior to admission hemograms were unknown and a comparison with admission hemograms was not possible; baseline conditions associated with immunomodulation or chronic leukocytosis were not ruled out.

## 6 CONCLUSIONS

NLr is an independent predictor of IHCs in patients admitted with TTS. Admission hemogram may be an easy and fast tool for risk stratification of IHCs in TTS.

## CONFLICT OF INTEREST

The authors declare no potential conflict of interest.

## AUTHORS CONTRIBUTION

All authors have read and approved the manuscript.

## References

[1] Dias A , Núñez Gil IJ , Santoro F , et al. Takotsubo syndrome: state‐of‐the‐art review by an expert panel–part 1. Cardiovasc Revasc Med. 2019;20(1):70‐79. 10.1016/j.carrev.2018.11.015 30528096

[2] Santoro F , Núñez Gil IJ , Stiermaier T , et al. Assessment of the German and Italian stress cardiomyopathy score for risk stratification for in‐hospital complications in patients with Takotsubo syndrome. JAMA Cardiol. 2019;4:892 10.1001/jamacardio.2019.2597. 31389988 PMC6686773

[3] Stiermaier T , Moeller C , Oehler K , et al. Long‐term excess mortality in takotsubo cardiomyopathy:predictors, causes and clinical consequences. Eur J Heart Fail. 2016;18:650‐656. 26990821 10.1002/ejhf.494

[4] Arbel Y , Finkelstein A , Halkin A , et al. Neutrophil/lymphocyte ratio is related to the severity of coronary artery disease and clinical outcome in patients undergoing angiography. Atherosclerosis. 2012;225:456‐460. 23040448 10.1016/j.atherosclerosis.2012.09.009

[5] Dentali F , Nigro O , Squizzato A , et al. Impact of neutrophils to lymphocytes ratio on major clinical outcomes in patients with acute coronary syndromes: a systematic review and meta‐analysis of the literature. Int J Cardiol. 2018;266:31‐37. 29887466 10.1016/j.ijcard.2018.02.116

[6] Madhavan M , Prasad A . Proposed Mayo Clinic criteria for the diagnosis of Tako‐Tsubo cardiomyopathy and long‐term prognosis. Herz. 2010;35:240‐243. 20582391 10.1007/s00059-010-3339-x

[7] Madhavan M , Rihal CS , Lerman A , Prasad A . Acute heart failure in apical ballooning syndrome (TakoTsubo/ stress cardiomyopathy): clinical correlates and Mayo Clinic risk score. J Am Coll Cardiol. 2011;57:1400‐1401. 21414539 10.1016/j.jacc.2010.10.038

[8] Hansson GK . Inflammation, atherosclerosis, and coronary artery disease. N Engl J Med. 2005;352:1685‐1695. 15843671 10.1056/NEJMra043430

[9] Bhat T , Teli S , Rijal J , et al. Neutrophil to lymphocyte ratio and cardiovascular diseases: a review. Expert Rev Cardiovasc Ther. 2013;11:55‐59. 23259445 10.1586/erc.12.159

[10] Simpson E , Cantor H . Regulation of the immune response by subclasses of T lymphocytes. II. The effect of adult thymectomy upon humoral and cellular responses in mice. Eur J Immunol. 1975;5:337‐343. 1086235 10.1002/eji.1830050509

[11] Brunetti ND , D'Antuono C , Rana M , D'Arienzo G , de Gennaro L , di Biase M . Lymphocyte subset characterization in patients with early clinical presentation of coronary heart disease. J Thromb Thrombolysis. 2012;34:475‐482. 22903683 10.1007/s11239-012-0761-3

[12] Brunetti ND . Hot stuff’: inflammatory lymphocyte populations in acute coronary syndrome. Cell Mol Immunol. 2015;12:513‐514. 25363525 10.1038/cmi.2014.96 PMC4496547

[13] Shen XH , Chen Q , Shi Y , Li HW . Association of neutrophil/lymphocyte ratio with long‐term mortality after ST elevation myocardial infarction treated with primary percutaneous coronary intervention. Chin Med J (Engl). 2010;123:3438‐3443. 22166528

[14] Ikeda U , Ikeda M , Oohara T , Kano S , Yaginuma T . Mitogenic action of interleukin‐1 alpha on vascular smooth muscle cells mediated by PDGF. Atherosclerosis. 1990;84:183‐188. 2282097 10.1016/0021-9150(90)90089-2

[15] Kim MH , Gorouhi F , Ramirez S , et al. Catecholamine stress alters neutrophil trafficking and impairs wound healing by β2‐adrenergic receptor‐mediated upregulation of IL‐6. J Invest Dermatol. 2014;134:809‐817. 24121404 10.1038/jid.2013.415 PMC4013292

[16] Núñez J , Fácila L , Llàcer A , et al. Prognostic value of white blood cell count in acute myocardial infarction: long‐term mortality. Rev Esp Cardiol. 2005;58:631‐639. 15970118

[17] Ommen SR , Gibbons RJ , Hodge DO , Thomson SP . Usefulness of the lymphocyte concentration as a prognostic marker in coronary artery disease. Am J Cardiol. 1997;79:812‐814. 9070569 10.1016/s0002-9149(96)00878-8

[18] Thomson SP , McMahon LJ , Nugent CA . Endogenous cortisol: a regulator of the number of lymphocytes in peripheral blood. Clin Immunol Immunopathol. 1980;17:506‐514. 7192197 10.1016/0090-1229(80)90146-4

[19] Cook‐Mills JM , Cohen RL , Perlman RL , Chambers DA . Inhibition of lymphocyte activation by catecholamines: evidence for a non‐classical mechanism of catecholamine action. Immunology. 1995;85:544‐549. 7558147 PMC1383781

[20] Aukrust P , Waehre T , Damås JK , Gullestad L , Solum NO . Inflammatory role of platelets in acute coronary syndromes. Heart. 2001;86:605‐606. 11711447 10.1136/heart.86.6.605 PMC1730032

[21] Furman MI , Benoit SE , Barnard MR , et al. Increased platelet reactivity and circulating monocyte‐platelet aggregates in patients with stable coronary artery disease. J Am Coll Cardiol. 1998;31:352‐358. 9462579 10.1016/s0735-1097(97)00510-x

[22] Li W , Liu Q , Tang Y . Platelet to lymphocyte ratio in the prediction of adverse outcomes after acute coronary syndrome: a meta‐analysis. Sci Rep. 2017;7:40426. 28071752 10.1038/srep40426 PMC5223131

[23] Dehghani MR , Rezaei Y , Taghipour‐Sani L . White blood cell count to mean platelet volume ratio as a novel non‐invasive marker predicting long‐term outcomes in patients with non‐ST elevation acute coronary syndrome. Cardiol J. 2015;22:437‐445. 25733319 10.5603/CJ.a2015.0015

[24] Pirzer R , Elmas E , Haghi D , et al. Platelet and monocyte activity markers and mediators of inflammation in Takotsubo cardiomyopathy. Heart Vessels. 2012;27:186‐192. 21416113 10.1007/s00380-011-0132-6

[25] Núñez‐Gil IJ , Bernardo E , Feltes G , et al. Platelet function in Takotsubo cardiomyopathy. J Thromb Thrombolysis. 2015;39:452‐458. 25052832 10.1007/s11239-014-1109-y

[26] Santoro F , Costantino MD , Guastafierro F , et al. Inflammatory patterns in Takotsubo cardiomyopathy and acute coronary syndrome: a propensity score matched analysis. Atherosclerosis. 2018;274:157‐161. 29783063 10.1016/j.atherosclerosis.2018.05.017

[27] Santoro F , Tarantino N , Ferraretti A , et al. Serum interleukin 6 and 10 levels in Takotsubo cardiomyopathy: increased admission levels may predict adverse events at follow‐up. Atherosclerosis. 2016;254:28‐34. 27680775 10.1016/j.atherosclerosis.2016.09.012

[28] Murakami T , Yoshikawa T , Maekawa Y , et al. Characterization of predictors of in‐hospital cardiac complications of takotsubo cardiomyopathy: multi‐center registry from Tokyo CCU network. J Cardiol. 2014;63:269‐273. 24139869 10.1016/j.jjcc.2013.09.003

[29] Stiermaier T , Santoro F , Graf T , et al. Prognostic value of N‐terminal pro‐B‐type natriuretic peptide in Takotsubo syndrome. Clin Res Cardiol. 2018;107:597‐606. 29675571 10.1007/s00392-018-1227-1

[30] Citro R , Bossone E , Parodi G , et al. Takotsubo Italian network investigators. Clinical profile and in‐hospital outcome of Caucasian patients with takotsubo syndrome and right ventricular involvement. Int J Cardiol. 2016;219:455‐461. 27387098 10.1016/j.ijcard.2016.06.039

[31] Uribarri A , Núñez‐Gil IJ , Conty DA , et al. Short‐ and long‐term prognosis of patients with Takotsubo syndrome based on different triggers: importance of the physical nature. J Am Heart Assoc. 2019;8:e013701. 31830875 10.1161/JAHA.119.013701 PMC6951081

[32] Zhang S , Diao J , Qi C , et al. Predictive value of neutrophil to lymphocyte ratio in patients with acute ST segment elevation myocardial infarction after percutaneous coronary intervention: a meta‐analysis. BMC Cardiovasc Disord. 2018;18:75. 29716535 10.1186/s12872-018-0812-6 PMC5930503

